# Supplementary material for: The potential impacts of vector host species fidelity on zoonotic arbovirus transmission
Source: PLoS Negl Trop Dis. 2025 May 8;19(5):e0012196. doi: 10.1371/journal.pntd.0012196 (PMC12061148; doi:10.1371/journal.pntd.0012196)
Supplement: S1 Text — A. Supplementary methods: A.1 Derivation of the basic reproduction number; A.2 Estimation of species-specific host preference and fidelity. B. Supplementary plots: mosquito feeding patterns under varying fidelity (Fig A), likelihood plot for fidelity estimation (Fig B), and global sensitivity analysis (Fig C). C. Supplementary tables: estimated host preference and fidelity values (Table A) and cumulative incidence of infected hosts (Table B). (PDF) [file pntd.0012196.s001.pdf]

S1 Text

A Supplementary methods

A.1 Derivation of basic reproduction number

Mathematically, R_0 is derived from the largest eigenvalue of the Next Generation Matrix (NGM) FV^{-1}, where:

$$F = \left[\frac{\partial \mathcal{F}_i}{\partial x_j}(x_0)\right], \quad \text{and} \quad V = \left[\frac{\partial \mathcal{V}_i}{\partial x_j}(x_0)\right], \quad 1 \leq j \leq m = 3.$$

Here, $F_i(x)$ represents the rate of new infections appearing in infectious compartment i and $V_i = V_i^- - V_i^+$ represents the net transition rate between compartment i and other infected compartments due to other means [1]. Specifically, $V_i^+(x)$ denotes the transfer rate into compartment i, while $V_i^-(x)$ denotes the transfer rate out of compartment i.

From the simple model in Equation 2, the disease-free equilibrium (DFE) is $(S^m, I^m, S_A, I_A, R_A, S_D, I_D, R_D) =$ $(N^m, 0, H_A, 0, 0, H_D, 0, 0)$. The Next Generation Matrix (NGM) approach [1] can be used to separate the infected equations into two matrices, $\mathcal{F}$ and $\mathcal{V}$ as follows:

$$\mathcal{F} = \begin{bmatrix} \rho_A \alpha \beta S^m \frac{I_A}{H_A} \\ \rho_A \alpha \beta I^m \frac{S_A}{H_A} \\ \rho_A \alpha \beta I^m \frac{S_D}{H_D} \end{bmatrix} \text{ and } \mathcal{V} = \begin{bmatrix} \mu_m I^m \\ (\gamma + \mu) I_A \\ (\gamma + \mu) I_D \end{bmatrix}.$$

Lineariasing the matrices at DFE, we get:

$$FV^{-1} = \begin{bmatrix} 0 & \rho_A \alpha \beta \frac{N^m}{H_A} & 0 \\ \rho_A \alpha \beta & 0 & 0 \\ \rho_D \alpha \beta & 0 & 0 \end{bmatrix} \begin{bmatrix} \frac{1}{\mu_m} & 0 & 0 \\ 0 & \frac{1}{(\gamma+\mu)} & 0 \\ 0 & 0 & \frac{1}{(\gamma+\mu)} \end{bmatrix} = \begin{bmatrix} 0 & \frac{\rho_A \alpha \beta N^m}{(\gamma+\mu) H_A} & 0 \\ \frac{\rho_A \alpha \beta}{\mu_m} & 0 & 0 \\ \frac{\rho_D \alpha \beta}{\mu_m} & 0 & 0 \end{bmatrix}$$

$$= \begin{bmatrix} 0 & \frac{\rho_A \alpha \beta N^m}{(\gamma+\mu) H_A} & 0 \\ \frac{\rho_A \alpha \beta}{\mu_m} & 0 & 0 \\ \frac{\rho_D \alpha \beta}{\mu_m} & 0 & 0 \end{bmatrix}$$

$$R_0 = \rho(FV^{-1}) = \sqrt{\frac{\rho_A \alpha \beta N^m}{(\gamma + \mu) H_A} \times \frac{\rho_A \alpha \beta}{\mu_m}} \qquad \text{(A)}$$

For the extended model in Equation 3, at DFE, $(S^m, S_A^m, S_D^m) = \big((1-\alpha)N^m, \rho_A \alpha N^m, \rho_D \alpha N^m\big)$, and the corresponding matrices $\mathcal{F}$ and $\mathcal{V}$ are:

$$\mathcal{F} = \begin{bmatrix} f_1 \alpha \beta \frac{I_A}{H_A} S_A^m + \rho_A \alpha \beta \frac{I_A}{H_A} S^m \\ f_2 \alpha \beta \frac{I_A}{H_A} S_D^m \\ f_1 \alpha \beta I_A^m \frac{S_A}{H_A} + f_2 \alpha \beta I_D^m \frac{S_A}{H_A} \\ f_3 \alpha \beta I_D^m \frac{S_D}{H_A} + f_4 \alpha \beta I_A^m \frac{S_D}{H_A} \end{bmatrix} \text{ and } \mathcal{V} = \begin{bmatrix} \mu_m I_A^m \\ \mu_m I_D^m \\ (\gamma + \mu) I_A \\ (\gamma + \mu) I_D \end{bmatrix},$$

with $f_1 = [f + (1-f)\rho_A]$, $f_2 = [(1-f)\rho_A]$, $f_3 = [f + (1-f)(1-\rho_A)]$, and $f_4 = [(1-f)(1-\rho_A)]$.

Compute the linearised matrices at DFE,

$$FV^{-1} = \begin{bmatrix} 0 & 0 & \frac{f_1 \alpha \beta \rho_A \alpha N^m + \rho_A \alpha \beta (1-\alpha) N^m}{H_A} & 0 \\ 0 & 0 & \frac{f_1 \alpha \beta \rho_D \alpha N^m}{H_A} & 0 \\ f_1 \alpha \beta & f_2 \alpha \beta & 0 & 0 \\ f_4 \alpha \beta & f_3 \alpha \beta & 0 & 0 \end{bmatrix} \begin{bmatrix} \frac{1}{\mu_m} & 0 & 0 & 0 \\ 0 & \frac{1}{\mu_m} & 0 & 0 \\ 0 & 0 & \frac{1}{(\gamma+\mu)} & 0 \\ 0 & 0 & 0 & \frac{1}{(\gamma+\mu)} \end{bmatrix}$$

$$
= \begin{bmatrix} 0 & 0 & \frac{f_1\alpha\beta\rho_A\alpha N^m + \rho_A\alpha\beta(1-\alpha)N^m}{(\gamma+\mu)H_A} & 0 \\ 0 & 0 & \frac{f_2\alpha\beta\rho_D\alpha N^m}{(\gamma+\mu)H_A} & 0 \\ \frac{f_1\alpha\beta}{\mu_m} & \frac{f_2\alpha\beta}{\mu_m} & 0 & 0 \\ \frac{f_4\alpha\beta}{\mu_m} & \frac{f_3\alpha\beta}{\mu_m} & 0 & 0 \end{bmatrix}
$$

$$
R_0 = \sqrt{\frac{f_1\alpha\beta\rho_A\alpha N^m + \rho_A\alpha\beta(1-\alpha)N^m}{(\gamma+\mu)H_A} \times \frac{f_1\alpha\beta}{\mu_m} + \frac{f_2\alpha\beta(1-\rho_A)\alpha N^m}{(\gamma+\mu)H_A} \times \frac{f_2\alpha\beta}{\mu_m}}. \tag{B}
$$

A.2 Estimation of species-specific initial host preference and fidelity

We estimated the initial preference for the amplifying host species (ρ_A) using data from a choice experiment conducted by Mwandawiro et al. [2]. In the experiment, naive mosquitoes (*Cx. tritaeniorhynchus*, *Cx. gelidus*, and *Cx. vishnui*) were released into a net containing a pig and a cow. Feeding was allowed for a specific period, after which all mosquitoes were recaptured, identified by species, and classified into fed and unfed [2]. Mosquitoes that were unfed or fed on both hosts were excluded from the analysis, as they were considered to have shown no preference. For each species, ρ_A was calculated as the proportion of mosquitoes that fed on a pig out of the total number that fed exclusively on either a pig or a cow. A binomial test was used to calculate 95% confidence intervals for ρ_A and to test whether the observed preference is significantly different from an equal preference for both hosts (null hypothesis: $\rho_A = 0.5$).

To estimate fidelity, f for each mosquito species, we used data from Mwandawiro et al. [2]. Mosquitoes previously imprinted on either pigs or cows were released into a net containing both animals. The subsequent host choices of the mosquitoes were recorded, including whether they switched hosts or remained loyal to their initial host species. We used a likelihood function to describe the probability of observing the data given fidelity f and the initial preference for the amplifying host (pigs, ρ_A). The likelihood is expressed as:

$$
\mathcal{L}(f|\text{data}) = \prod_{i\in\{A,D\}} \binom{n_i}{r_i} [f + (1-f)\rho_i]^{r_i} \, [(1-f)\rho_{\neg i}]^{n_i - r_i} \,. \tag{C}
$$

Here, n_i is the total number of mosquitoes imprinted on host species i, r_i is the number of mosquitoes imprinted on host species i that fed on host species i, ρ_i is the initial preference for host species i, and $\rho_{\neg i} = 1 - \rho_i$ is the initial preference for alternative host species. Fidelity values were estimated by minimising the negative log-likelihood function using the “optim” function in R [3], and confidence intervals for f were derived from the Hessian matrix of the optimised likelihood function.

B Supplementary figures

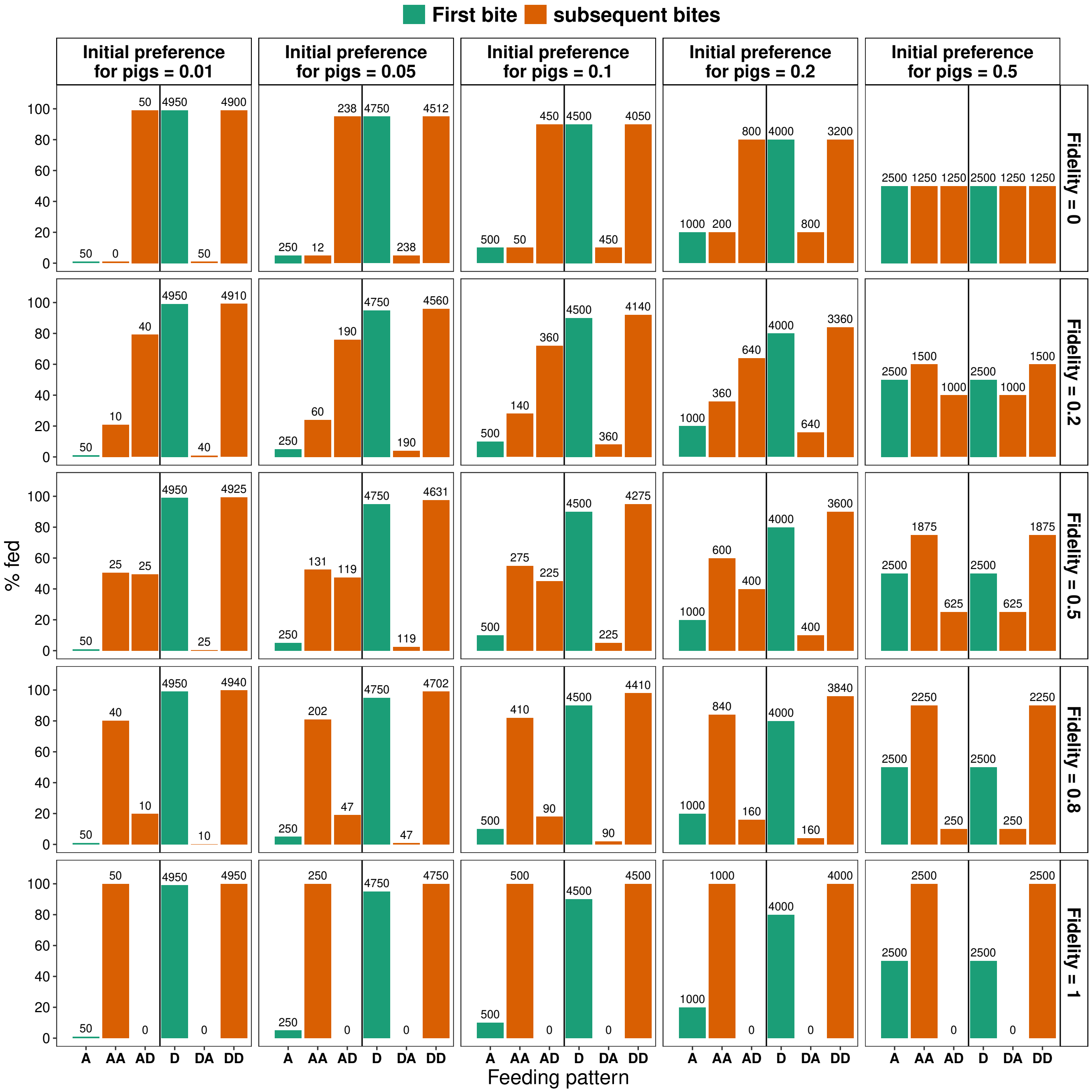

Figure A: Variation of feeding patterns of mosquitoes with fidelity, in the instance where cows (C) and pigs (P) are available in equal proportions. The height of the bars indicates the proportion of mosquitoes that fed on host species (x-axis), with their corresponding numbers at the top. The fill indicates the proportion of first and subsequent bites. The top panel represents an increasing initial preference for pigs (left-to-right), while the right panel represents an increasing fidelity (top-to-bottom).

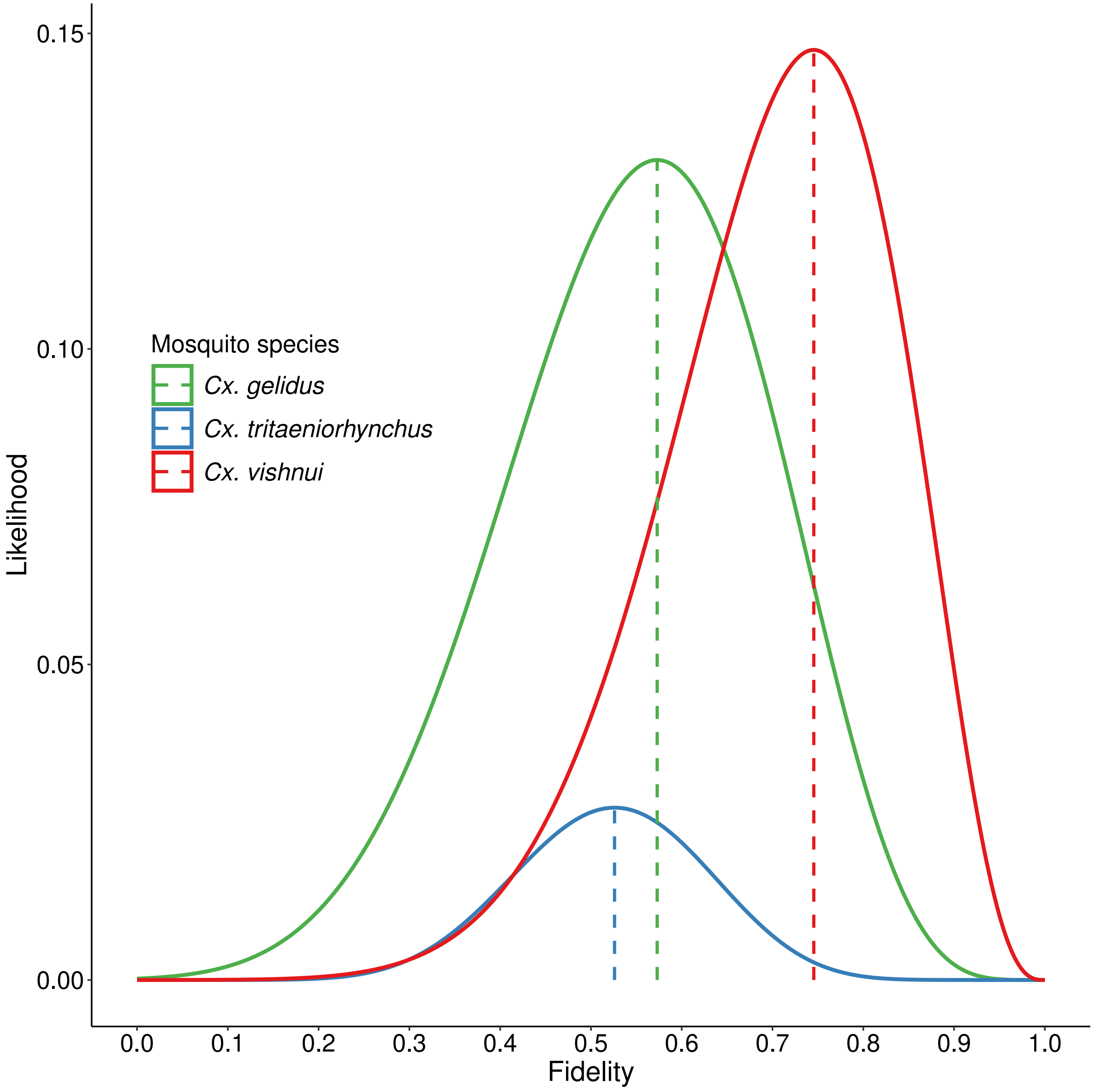

Figure B: Fidelity estimates of three JE mosquito species. Based on the experiment conducted by Mwandawiro et al. [2], we estimated the initial preference for amplifying hosts for three mosquito species competent for JEV: *Cx. tritaeniorhynchus*, *Cx. gelidus*, and *Cx. vishnui*. Fidelity values were subsequently estimated using maximum likelihood estimation (MLE) from biting behaviour data, as described in Figure 1. The resulting fidelity estimates (f) are presented in Table A with their corresponding 95% confidence intervals.

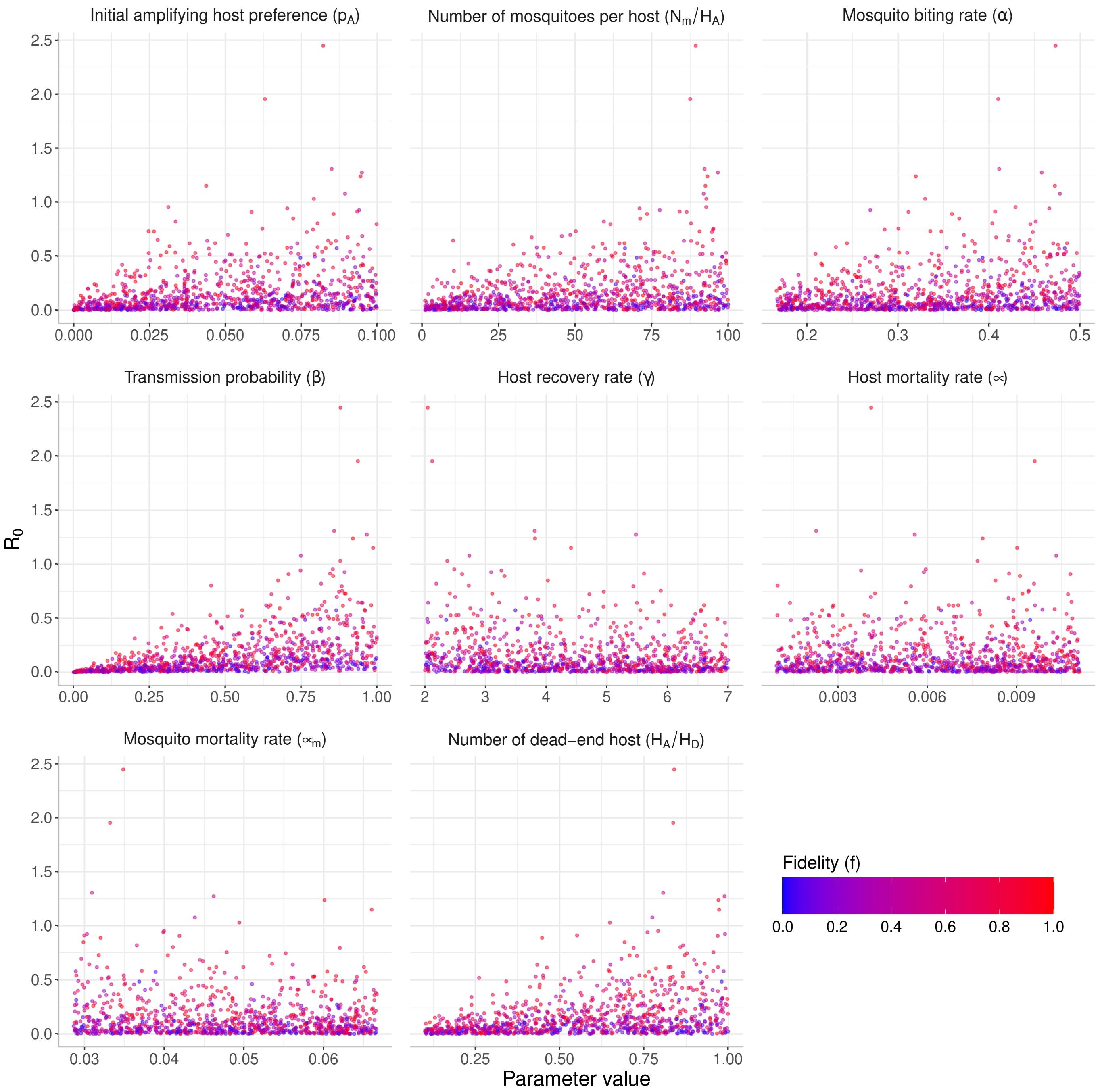

Figure C: Global sensitivity analysis. To show the variability of R_0 with respect to all parameter ranges, we used random Latin hypercube design to simulate $1\,000$ parameter sets with the ranges of parameters from Table 3, which were then used to calculate R_0 values.

C Supplementary tables

Table A: Estimates of initial preference for the amplifying host species and fidelity for three JE vectors (*Cx. tritaeniorhynchus*, *Cx. gelidus*, and *Cx. vishnui*), including 95% confidence intervals.

Species	Initial preference (95% CI)	Fidelity estimate (95% CI)
Cx. tritaeniorhynchus	0.05 (0.00, 0.31)	0.53 (0.31, 0.74)
Cx. gelidus	0.22 (0.13, 0.34)	0.57 (0.26, 0.88)
Cx. vishnui	0.15 (0.02, 0.45)	0.75 (0.49, 1.00)

Table B: Cumulative incidence of infected amplifying and dead-end host assuming 5 mosquitoes per host and fixed parameter values in Table 3.

Scenario			Cumulative incidence	
Preference for competent hosts	Fidelity	Proportion of competent hosts	Competent hosts	Dead-end hosts
0.05	0.0	0.1	0.02	4.09
		0.5	0.37	6.84
	0.2	0.1	1.20	9.02
		0.5	6.58	48.91
	0.5	0.1	33.47	106.26
		0.5	326.92	396.15
	0.8	0.1	64.34	93.15
		0.5	390.11	333.88
	1.0	0.1	74.27	0.00
		0.5	432.14	0.00
0.20	0.0	0.1	0.31	11.01
		0.5	414.64	476.96
	0.2	0.1	55.64	563.93
		0.5	434.60	478.19
	0.5	0.1	82.95	537.84
		0.5	461.44	472.44
	0.8	0.1	92.57	389.27
		0.5	476.74	445.88
	1.0	0.1	94.92	0.00
		0.5	482.81	0.00

References

1. van den Driessche P, Watmough J. Reproduction numbers and sub-threshold endemic equilibria for compartmental models of disease transmission. Mathematical Biosciences. 2002;180(12):2948. doi:10.1016/s0025-5564(02)00108-6.
2. Mwandawiro C, Boots M, Tuno N, Suwonkerd W, Tsuda Y, Takagi M. Heterogeneity in the host preference of Japanese encephalitis vectors in Chiang Mai, northern Thailand. Transactions of the Royal Society of Tropical Medicine and Hygiene. 2000;94(3):238242. doi:10.1016/s0035-9203(00)90303-1.
3. R Core Team. R: A Language and Environment for Statistical Computing; 2024. Available from: `https://www.R-project.org/`.
