# Supplementary material for: The potential impacts of vector host species fidelity on zoonotic arbovirus transmission
Source: PLoS Negl Trop Dis. 2025 May 8;19(5):e0012196. doi: 10.1371/journal.pntd.0012196 (PMC12061148; doi:10.1371/journal.pntd.0012196)
Supplement: Attachment — Submitted filename: Response_to_Reviewers.pdf [file pntd.0012196.s003.pdf]

## Response to Reviewers' Comments:

We thank the reviewers and editor for their constructive comments and we provide a point by point response in the text below.

### Methods

- **Is the study design appropriate to address the stated objectives?**

**Comment:** I have a concern with the chosen compartmentalization of susceptible mosquitoes. I'm not sure why there are five categories of mosquitoes in the model - I think its more about tractability, but I'm having a hard time finding the biology in it (especially the infected mosquito fed on dead end host compartment). The nulliparous compartment makes sense, but then to me its not clear how things are moving through the system.

If a mosquito is only infected by the amplifying hosts (pigs), then I think the objective of fidelity would be to model individuals who take a first blood meal on a cow who then take a $1 + n$ blood meal on a pig. The infected compartment would then also represent a mix of individuals who became infected on their first bite + those that eventually become infected on blood meal $1 + n$. Once a mosquito is infected (either from the first or second blood meal) it would remain infected, correct?

Delta(nulliparous) = remains as written

Delta(susceptible but blood fed) = (proportion nulliparous fed on an uninfected pig) + (proportion nulliparous/parous fed on any cow) - (proportion susceptible but blood fed who then take a $1 + n$ blood meal on an infected pig)

Delta(infected, blood fed) = (proportion nulliparous fed on an infected pig) + (proportion susceptible but blood fed who then take a 1+n blood meal on an infected pig)

To me, and based on my translation from Figure 1 (which clearly displays the individual-level process of blood feeding) to equation 3 (which presents a population level process), fidelity only weighs that $1 + n$ blood meal.

Please clarify these concerns.

**Reply:** Thank you for your comment and the opportunity to clarify our approach. Your understanding of the model is exactly correct, in that fidelity comes into effect from the second feed onwards, and that the five compartments are for labelling mosquitoes imprinted onto different species (one nulliparous, two sets of SI states for the two imprinted host species).

As the infection pressures differ depending on whether the mosquito is nulliparous/imprinted on cow/imprinted on pig, it is necessary to divide the compartments this way in order to incorporate preference and fidelity. We agree however that as this is non-standard, we should make our approach clearer. To begin, we have added additional text to the introduction to draw the difference between

host preference (which dictates the first feed) and fidelity (for feeds thereafter). We have also added to the subsection *A vector-borne pathogen model with host preference and fidelity* some additional text explaining the need for five compartments.

On the biology of the five compartments, we have tried to flesh out this explanation in the method to make it clearer. We state from line 134: 'To generalise Equation 2 to explore fidelity, we need to label mosquitoes based on the host (if any) they are imprinted on to. We therefore label mosquitoes as either nulliparous (yet to take a blood meal) (Sm), imprinted on an amplifying host (SmA, or ImA if infected), and imprinted on a dead-end host (SmD , ImD if infected), bringing a total of five compartments. This allows us to keep track of mosquito activity once they have imprinted, for example, the compartment ImD allows for mosquitoes that initially imprinted on a cow, but by probability $(1 - f)\rho_A$, may fed on a pig and potentially become infected, however they still have probability $[f + (1 - f)\rho_D]$ of feeding on a cow subsequently.'

### Conclusions

- Are the limitations of analysis clearly described?

**Comment:** Yes though the model also shows vector abundance can overcome limitations of preference/fidelity though this is not mentioned in the discussion or results.

**Reply:** In the discussion on line 236, we have written: 'While our analysis has shown that, by incorporating fidelity, *Cx. tritaeniorhynchus* may be less important to transmission, we acknowledge this is when assuming that all vector species are present in equal abundance. In many times and places, *Cx. tritaeniorhynchus* may be substantially more abundant than other vector species and thus still be the most important vector, despite lower levels of fidelity to amplifying hosts'.

## Editorial and Data Presentation Modifications

Use this section for editorial suggestions as well as relatively minor modifications of existing data that would enhance clarity. If the only modifications needed are minor and/or editorial, you may wish to recommend "Minor Revision" or "Accept".

- Reviewer #2: I found myself conflating definitions of preference, fidelity, (vector) memory, and imprinting. Preference is rightfully the genetic basis of host choice (the probability the mosquito a priori chooses a host). Fidelity - defined as imprinting, makes sense too but that definition is presented too late in the manuscript. The distinction that fidelity only comes into play AFTER initial blood feeding could be a lot clearer in the report as well.

  The introduction is a bit bumpy - meaning the flow of ideas sometimes is not as concise as it could be. For instance, over twenty lines are given to describing a single, prior published paper? Also, some paragraphs are only 1 - 2 sentences.

**Reply:** We have added clear definitions of the three terms at the start of the section *"A vector-borne pathogen model with host preference and fidelity"*.

We have now focussed the introduction section only on papers concerning specifically mosquito memory. We have removed reference to the last two modelling studies that previously took up a substantial part of the second half of the introduction as they are not strictly about mosquito memory.

## Summary and General Comments

**Reviewer #1:** The authors present a mathematical model to explore the role of host fidelity in mosquito-borne disease transmission, using Japanese encephalitis virus as an example. The article is well written. The conclusions follow logically from the model.

I have only minor comments:

- The manuscript side-steps another potential mechanism for increasing amplification where amplifying hosts are less common and less preferred: local spatial heterogeneity. Even if pigs are outnumbered 140:1 at the regional scale, pigs may be locally concentrated and might outnumber cattle at the scale of an individual farm

  **Reply** From line 266 of the Discussion we state "...In addition, there are also spatial heterogeneities, including in host community composition, that likely affect transmission. Here our point is to highlight the potential role of fidelity and for this to be considered as a potential driver of transmission in regions where host community composition would, in the first instance, suggest that transmission is not possible."

- Range for gamma in Table 3 is incorrect – should be fractions as for the other parameters.

  **Reply:** We agree with the reviewer. This has been addressed in Table 2.

- The citation for the biting rate estimate was for Cx. quinquefasciatus. Is there a reason this species was chosen? I do not have access to the paper, to see if it provides biting rates for additional species. Taking a blood meal every 3 days strikes me as a fast turn-around for Culex, although biting rate is influenced by temperature. Not a major concern though, given the lack of sensitivity to biting rate in the sensitivity analysis.

  **Reply:** The cited article estimates the biting rate for Cx. quinquefasciatus only, with oviposition rates of $3-6$ days. We assumed a biting rate range corresponding to this oviposition range to align with other JEV modelling studies (e.g., `doi.org/10.1371/journal.pntd.0010572`, `doi.org/10.1371/journal.pone.0201209`). We acknowledge that this range is slightly faster than the estimates provided by Malainual et al., who reported gonotrophic cycle lengths of $5-8$ days for Cx. tritaeniorhynchus and Cx. gelidus under field conditions (`doi.org/10.7601/mez.49.105`). However, as you rightly

pointed out, our sensitivity analysis shows the model analysis is not greatly affected by variation in biting rates.

- Figure 4: A supplemental table with the total numbers infected would be helpful (or the numbers placed in the whitespace in the graph). I couldn't quite tell which resulted in more infected overall – a longer lower peak or the sharper higher peaks.

  **Reply:** Thank you for your comment. This has been added in Table B in S1 Text

- Figure S2: Is the y-axis label correct? Isn't this something to do with the likelihood of a given fidelity value? The methods used to calculate the fidelity estimates also come across as somewhat opaque to me – I'm not following the methods used here, except in the broadest sense.

  **Reply:** We have added a short section that describes the approach used. See Section A.2 in S1 Text.

**Reviewer #2:** In general, the manuscript investigates how blood meal imprinting (i.e., learned blood feeding) alters JEV transmission dynamics and then estimates vectoring potential of three important vector species. This is an interesting topic and relevant to the field of JEV and other VBD systems/models.

My primary scientific concern is that the fidelity-based ODEs, to me, don't match the biologically relevant stages of vectors - fidelity as presented in the manuscript should be more about tracking the development of infections in those species that initially blood fed on a dead end host, and simpler Susceptible/Infected classes of vectors may be just as efficient (and realistic) as those developed by the authors. My primary editorial concern is a stronger, clearer definition of fidelity is needed sooner in the manuscript and language needs to be more consistent between preference, fidelity, and imprinting.

- Line 37: only anopheles (the genus) is listed (?)

  **Reply:** We have added 'spp.' (Line 43) as the study involved multiple mosquito species. Listing them here individually isn't necessary for the point of the sentence.

- Lines 174 - 183 should come sooner in the methods

  **Reply:** Thank you for your suggestion. The content in the section 'Host choice experiment' is part of the results section rather than the methods. However, we have reviewed the methods section to provide context in Lines 146-148.

- Table 3: Mwandawiro 1999/2000 are foundational papers for this model. What were their estimates of fidelity - i.e., whats a realistic range of fidelity?

**Reply:** Mwandawiro 2000 did not provide a direct estimate of mosquito fidelity in their manuscript. However, we inferred fidelity values based on the likelihood of observed data from their experiment. See S1 Text.

- Lines 262 - 268: no citations?

  **Reply:** Thank you. Citations have now been added to lines 232 and 241.

- Discussion of cattle and malaria extensions - I think an important caveat is that feeding on cattle (in amplification outcomes) generates increased vector abundance such that while the fraction of the infected population is small, the overall population is enormous!. In this instance, fidelity would lead to perhaps non-linear increases in vector abundance (and a secondary outcome would be nonlinear changes in transmission)

  **Reply:** In the discussion on line 253 we have added 'in particular to quantify how fidelity may also cause increased vector density and subsequent effects of both altered mosquito to host ratio and host choice on transmission.'

## Additional Editor Comments:

- The analyses presented in this manuscript depend on the code developed, and use of a private Github repository is inadequate to ensure availability and reproducibility of the findings. Please include the code as supplementary information with the manuscript or post the code on a public, permanent repository. In addition to the reviewers' feedback, a PDF is attached with editorial comments.

  **Reply:** GitHub repository is now publicly accessible through `https://github.com/tijanisulaimon/mosquito-fidelity-model`.

- L31 - Replace "when the preferred host is also the host with highest competence" with "when the preference for highly competent hosts is sufficiently strong to overcome any losses in transmission to less-competent species"

  **Reply:** Done

- L33 - Replace "when a vector does not have an underlying preference" with "Or even if the preference for the competent host is relatively weak"

**Reply:** This sentence now reads '...using a general modelling framework, showed that higher host diversity can result in amplification of a pathogen when the preference for highly competent host species is sufficiently strong to overcome any losses in transmission to less-competent species. However, diversity can cause dilution when a vector prefers host species that have lower competence, or when a vector does not have an underlying preference for any specific host species' (Line 35)

- L38 - Replace "Ginsberg et al. (2021) showed that ..." deviates from the paragraph's topic of host diversity vs. transmission

  **Reply:** Deleted

- L154: Is there any potential for negative experience and reduction in affinity for a host following a first blood meal?

  **Reply** We state in the introduction on line 55 that there is evidence that both attraction and aversion behaviour can be learned when mosquitoes are exposed to a particular odour alongside either a positive or negative experience. We then describe how the modelling of fidelity encompasses both processes in the fidelity term - as it could be driven by increased aversion to the more-defensive host, increased attraction to the less-defensive host, or both.
